# Proliferation and Glia-Directed Differentiation of Neural Stem Cells in the Subventricular Zone of the Lateral Ventricle and the Migratory Pathway to the Lesions after Cortical Devascularization of Adult Rats

**DOI:** 10.1155/2016/3625959

**Published:** 2016-05-11

**Authors:** Feng Wan, Hua-Jing Bai, Jun-Qi Liu, Mo Tian, Yong-Xue Wang, Xin Niu, Yin-Chu Si

**Affiliations:** ^1^Department of Anatomy, School of Basic Medical Sciences, Beijing University of Chinese Medicine, Beijing 100029, China; ^2^Dongzhimen Hospital, Beijing University of Chinese Medicine, Beijing 100700, China; ^3^International School, Beijing University of Chinese Medicine, Beijing 100029, China

## Abstract

We investigated the effects of cortical devascularization on the proliferation, differentiation, and migration of neural stem cells (NSCs) in the subventricular zone (SVZ) of the lateral ventricle of adult rats. 60 adult male Wistar rats were randomly divided into control group and devascularized group. At 15 and 30 days after cerebral cortices were devascularized, rats were euthanized and immunohistochemical analysis was performed. The number of PCNA-, Vimentin-, and GFAP-positive cells in the bilateral SVZ of the lateral wall and the superior wall of the lateral ventricles of 15- and 30-day devascularized groups increased significantly compared with the control group (*P* < 0.05 and *P* < 0.01). The area density of PCNA-, Vimentin-, and GFAP-positive cells in cortical lesions of 15- and 30-day devascularized groups increased significantly compared with the control group (*P* < 0.05 and *P* < 0.01). PCNA-, GFAP-, and Vimentin-positive cells in the SVZ migrated through the rostral migratory stream (RMS), and PCNA-, GFAP-, and Vimentin-positive cells from both the ipsilateral and contralateral dorsolateral SVZ (dl-SVZ) migrated into the corpus callosum (CC) and accumulated, forming a migratory pathway within the CC to the lesioned site. Our study suggested that cortical devascularization induced proliferation, glia-directed differentiation, and migration of NSCs from the SVZ through the RMS or directly to the corpus callosum and finally migrating radially to cortical lesions. This may play a significant role in neural repair.

## 1. Introduction

Cerebral ischemic stroke is a leading cause of human death and disability [1–3]. Stroke and traumatic brain injury lead to cell death, characterized by a loss of neurons and glial cells within the brain [4, 5]. In the early 1990s, self-replicating neural stem cells (NSCs) were identified in the central nervous system (CNS). These cells proliferate, migrate, and differentiate into all the cell types of the brain and spinal cord, including neurons, astrocytes, and oligodendrocytes [6–8]. This discovery brought new hope for the patients with central degenerative diseases and injuries.

In adult mammals, endogenous NSCs mainly exist in two regions of the brain: the subventricular zone (SVZ) by the lateral ventricles and the subgranular zone (SGZ) of the dentate gyrus in the hippocampus, where the microenvironment is beneficial for the survival of NSCs [9, 10]. NSCs in the SVZ proliferate and migrate tangentially along the rostral migratory stream (RMS) to the olfactory bulb (OB), where they differentiate into either granule cells (GC) or periglomerular cells (PG) [11]. NSCs in the SGZ proliferate and migrate to the granular cell layers and then differentiate into new granule cells [12]. Study also found that NSCs in fetal brain have distinct morphologies and are widely distributed in the hippocampus, subventricular zone, striatum, and cortex, the amount of NSCs reduces over gestational age, and these stem cells can differentiate into locally required cerebral nerve cells [13, 14]. Upon injury, NSCs in a resting state activate, proliferate, migrate to the injured site, and differentiate into new nerve cells. These new cells can replace injured cells, participate in the formation of new neural circuits, and promote the structural and functional repair of the brain damage [15].

In this study, we applied immunohistochemical (IHC) staining of PCNA, GFAP, and Vimentin to measure proliferation and glia-directed differentiation of NSCs in the SVZ and the cortical lesion site and further traced the migratory path from the SVZ to lesions. We observed proliferation and glia-directed differentiation of NSCs in the SVZ and lesions in adult rat brains after cortical devascularization. NSCs from the SVZ through the RMS or directly to the corpus callosum after cortical devascularization formed migration flows and dispersed to cortical lesions.

## 2. Materials and Methods

### 2.1. Experimental Animals and Groupings

60 adult male Wistar rats, grade SPF/MF, were provided by the Department of Zoology, Peking Union Medical College. Rats weighing from 200 g to 250 g were provided sterilized water and food in the Animal Laboratory of Science Experimental Center, Beijing University of Chinese Medicine. All rats were randomly divided into control group (30 rats) and experimental group (30 rats). The study protocol was approved by the Joint Ethical Review Committee of the Beijing University of Chinese Medicine (number R-20130303-5). All surgery was performed under chloral hydrate anesthesia, and all efforts were made to minimize suffering.

### 2.2. Model Establishment

According to previous report [16], rats were anesthetized with 100 g/L chloral hydrate (400 mg/kg body weight) before opening the left skull to expose the dura mater and soft meninges. The left cortical blood vessels were gently wiped with physiologic saline until the vessels could not be seen under an anatomical microscope. The wiped left side was the experimental side. The skin was sutured at the end of the surgery. Rats survived for 15 days and 30 days after surgery, respectively.

### 2.3. Material Preparation

At 15 days (15 rats in control group and 15 rats in model group) and 30 days (15 rats in control group and 15 rats in model group) after surgery, rats in each group were anesthetized with 100 g/L chloral hydrate and then quickly decapitated and brain tissue was fixed with 30% neutral-buffered formalin, dehydrated, and embedded in paraffin. Continuous coronal or sagittal sections were sliced using a microtome at a thickness of 6 *μ*m.

### 2.4. Immunohistochemical Assay

The ABC method was used to stain 6 *μ*m paraffin-embedded sections. Paraffin was removed by xylene treatment twice for 5 minutes and then rehydrated with 100%, 95%, 90%, 80%, and 70% graded ethanol to distilled water, with two washes per step. Slices were then washed three times with 0.01 mol/L PBS (pH 7.2), placed in 10 g/L H_2_O_2_ (methanol configuration) for 30 minutes at room temperature to eliminate endogenous peroxidase, and washed three more times. Then, the sections were digested with Proteinase K for 20 minutes at 37°C, followed by three more washes. The sections were then blocked for 30 minutes at 37°C with 100 g/L normal goat serum, incubated with the primary antibody (mouse anti-Vimentin antibody, Santa Cruz, sc-6260; mouse anti-PCNA antibody, Abcam, ab29; rabbit anti-GFAP antibody, Abcam, ab48050) in 0.01 mol/L PBS for 72 h at 4°C. The sections were washed three times and incubated with biotinylated secondary antibody working solution and then HRP-Streptavidin working solution (S-A/HRP, ZYMED) for 2 h each at 37°C. After washing, DAB (0.5 g/L DAB: 0.1 g/L H_2_O_2_, 0.01 mol/L PBS) was added and incubated for 5 min at room temperature. The sections were dehydrated, cleared, and coverslipped. Negative control received the same treatment except that the primary antibody was replaced with 0.01 mol/L PBS.

### 2.5. Image Analysis and Statistical Methods

The number of PCNA-, Vimentin-, and GFAP-positive cells in the SVZ and the area density of positive cells in the cortical lesion site were analyzed using Image-Pro Plus 6.0 software. Statistical analyses were conducted using SPSS 17.0 statistical software. Quantitative data were presented as mean ± standard deviation values and groups' comparison using *t*-test. *P* < 0.05 was taken as statistically significant.

## 3. Results

### 3.1. Distribution of Proliferation and Migration of NSCs in the SVZ after Cortical Devascularization

In normal adult rats, PCNA-, GFAP-, and Vimentin-positive cells were mainly distributed in the lateral wall of the anterior corner of the lateral ventricle, especially in the dorsal wall of the lateral ventricle. Cells extended outward to form a dorsolateral corner (dlC), which appeared triangular in the coronal plane. These positive cells in the dlC extended outward between the corpus callosum and the caudate putamen to form a lateral extension (LE) with a flat ribbon-like or laminar structure.

**Figure 1:**
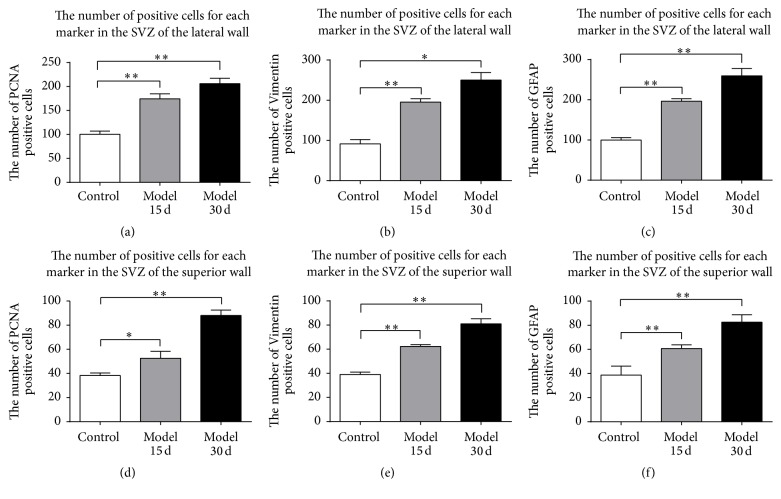
The number of positive cells for each marker in the SVZ after cortical devascularization. (a) The number of PCNA-positive cells in the SVZ of the lateral wall. (b) The number of Vimentin-positive cells in the SVZ of the lateral wall. (c) The number of GFAP-positive cells in the SVZ of the lateral wall. (d) The number of PCNA-positive cells in the SVZ of the superior wall. (e) The number of Vimentin-positive cells in the SVZ of the superior wall. (f) The number of GFAP-positive cells in the SVZ of the superior wall. ^*∗*^ *P* < 0.05 and ^*∗∗*^ *P* < 0.01, compared to control group.

**Figure 2:**
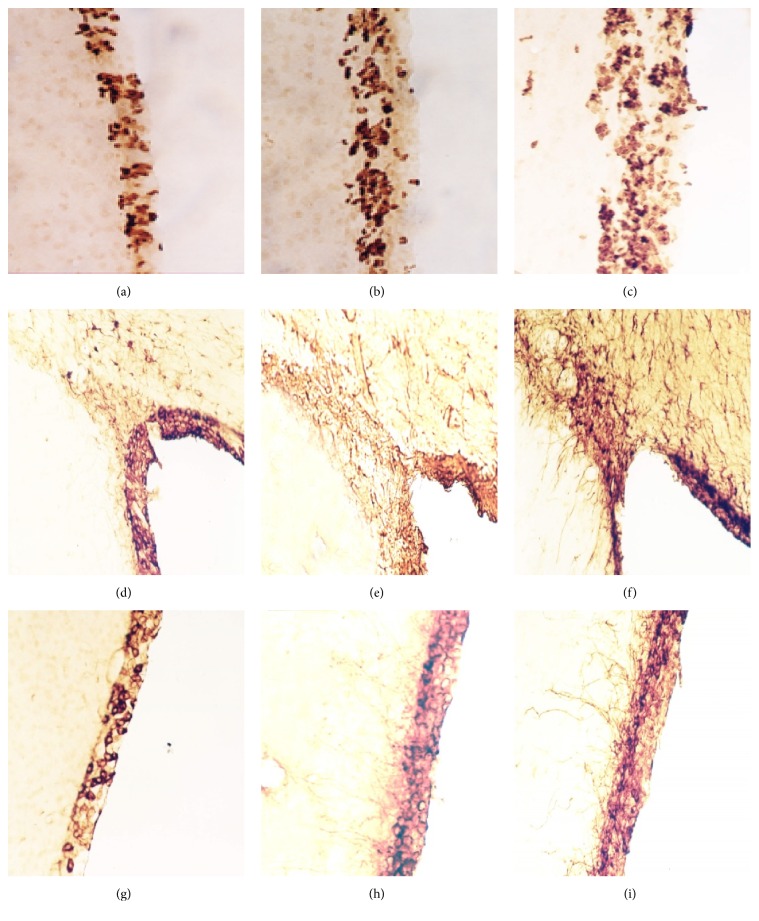
PCNA-, Vimentin-, and GFAP-positive cells in the SVZ of the lateral ventricle (100x). (a) PCNA-positive cells in the SVZ of control groups. (b) PCNA-positive cells in the SVZ of devascularized groups at 15 days. (c) PCNA-positive cells in the SVZ of devascularized groups at 30 days. (d) Vimentin-positive cells in the SVZ of control groups. (e) Vimentin-positive cells in the SVZ of devascularized groups at 15 days. (f) Vimentin-positive cells in the SVZ of devascularized groups at 30 days. (g) GFAP-positive cells in the SVZ of control groups. (h) GFAP-positive cells in the SVZ of devascularized groups at 15 days. (i) GFAP-positive cells in the SVZ of devascularized groups at 30 days.

Compared with the control group, the number of PCNA-, Vimentin-, and GFAP-positive cells in the SVZ of the contralateral and ipsilateral dlC (including the dorsal wall) increased in model group (Figures 1 and 2). Areas of positive cells expanded after cortical devascularization. Positive cells migrated into the corpus callosum and formed a radial migration path to cortical lesions. The SVZ of the dlC, the LE, and the dorsal wall are collectively known as the dorsolateral subventricular zone (dl-SVZ). Cortical devascularization caused positive cells in the dl-SVZ to migrate from the dorsal side of the lateral wall and the superior wall to the dlC. From there, cells migrated from the dlC to the LE, and then cells formed radiating cell migratory chains from the inside to the outside of the dl-SVZ in the coronal plane.

### 3.2. Effects of Cortical Devascularization on the PCNA-Positive Cells in the SVZ of the Lateral Ventricle

In normal adult rats, PCNA-positive cells were mainly distributed in the SVZ in the lateral wall of the anterior corner of the lateral ventricle, and PCNA-positive cells entered the RMS or the corpus callosum from the dlC of the lateral ventricle. PCNA-positive cells are also expressed in the ependymal layer, but the amount and the strength are lower than those in the SVZ.

After 15 days and 30 days of cortical devascularization, PCNA-positive cells in the anterior corner of the lateral wall of the SVZ proliferated and appeared in a split phase. Nuclei exist in pairs with rhabditiform or ellipsis shape and multiple nuclei together into a cluster. Numbers of PCNA-positive cells increased and entered the RMS and the corpus callosum from the SVZ, migrating to the cortical lesions. Compared with the control group, the number of PCNA-positive cells in the SVZ of the lateral wall and the superior wall of the anterior corner of the lateral ventricle at 15 days and 30 days after cortical devascularization increased significantly (*P* < 0.05 and *P* < 0.01) (Figures 1(a), 1(d), 2(a), 2(b), and 2(c)).

### 3.3. Effects of Cortical Devascularization on Vimentin-Positive Cells in the SVZ of the Lateral Ventricle

In normal adult rats, Vimentin-positive cells are distributed in the SVZ of the anterior corner of the lateral ventricle, including the lateral wall, the superior wall, and the medial wall. In positive cells, Vimentin is expressed in the cytoplasm and cell processes. Most of the cell bodies were round or ellipsoid, with multiple processes of varying lengths. Vimentin-positive cells entered the corpus callosum from the SVZ of the superior wall and the RMS, which was formed by positive cells in the SVZ of the anterior corner. In the central part and the bottom corner of the lateral ventricles, there were more regularly arranged Vimentin-positive cells in the SVZ of the lateral wall, from which Vimentin-positive cells entered the lateral corpus callosum and began migration.

After 15 days and 30 days of cortical devascularization, Vimentin-positive cells significantly increased in the SVZ of the lateral ventricle and were amplified to multiple layers, especially in the lateral wall of the lateral ventricle. Morphology of Vimentin-positive cells changed significantly. Cells had more and thicker processes, and positive fibers entered the lateral corpus striatum and septal area from the SVZ of the lateral wall and the medial wall of the anterior corner. Vimentin-positive astrocytes entered into the corpus callosum from the SVZ, increased significantly, and migrated to the lesions. There was a larger increase in the number of Vimentin-positive cells in the SVZ at 30 days after cortical devascularization than at 15 days, and these cells migrated to the corpus callosum from the SVZ. Compared with the control group, there were more Vimentin-positive cells in the SVZ of the lateral wall and the superior wall of anterior corner in the model groups at 15 days and 30 days (*P* < 0.05 and *P* < 0.01) (Figures 1(b), 1(e), 2(d), 2(e), and 2(f)).

### 3.4. Effects of Cortical Devascularization on Expression of GFAP-Positive Cells in the SVZ of the Lateral Ventricle

In normal adult rats, the expression of GFAP-positive cells in the SVZ of the lateral ventricle decreased from rostral to caudal. Large numbers of GFAP-positive cells gathered in the anterior corner of the SVZ, with more in the rostral anterior corner where GFAP-positive cells left to form the RMS. GFAP-positive cell processes in the RMS show directionality, appearing before and after successive cells in migration chains. Few GFAP-positive cells were observed in the SVZ of the middle or the lower segment of the lateral walls. The expression of GFAP in the superior wall was mainly confined to the SVZ, where it connected with the lateral corner of the RMS, but the number of cells was low. GFAP-positive cells were not present in the SVZ of the medial wall of the lateral ventricle. From rostral to caudal in the lateral ventricle, the expression of GFAP gradually decreased from the central to the inferior corner of the lateral ventricle. Compared with GFAP-positive cells in the anterior corner of the lateral ventricle, GFAP-positive cells in the inferior corner of the lateral ventricle were smaller and more variable cell shapes.

At 15 days after cortical devascularization, the number of GFAP-positive cells significantly increased in the SVZ of the anterior corner of the lateral ventricle. GFAP-positive cells in the SVZ of the upper part of the lateral wall expanded to form multiple layers, mostly round and oval with many protrusions. GFAP-positive cells in the lateral corner were significantly amplified, and the GFAP-positive cells in the SVZ of the superior wall of the lateral ventricle notably increased as well. At 30 days after cortical devascularization, in comparison with the 15-day group, the number of GFAP-positive cells in the SVZ of the lateral wall and the superior wall of anterior corner of lateral ventricle increased and the reaction was enhanced. GFAP-positive cells were amplified in the SVZ of the superior lateral wall, the volume of cells became larger, the cell processes were more obvious, and the cells had numerous protrusions. The RMS, formed by GFAP-positive cells from the lateral corner, became significantly larger. Numerous GFAP-positive cells, which were emitted from the RMS at the lateral corner and the SVZ of the superior wall, entered the callosum and migrated to the cortical lesions. GFAP-positive cells significantly increased in cortical lesions as well as the surrounding cortex, the volume of cells enlarged, the percentage of positive cells with cell processes increased, and the cell processes were more apparent. Compared with the control group, GFAP-positive cells in the SVZ of the lateral wall and the superior wall of the anterior corner increased significantly in the devascularized groups at 15 days and 30 days (*P* < 0.05 and *P* < 0.01) (Figures 1(c), 1(f), 2(g), 2(h), and 2(i)).

### 3.5. Ratio of Glia Differentiated from NSCs in the SVZ of the Lateral Ventricle after Cortical Devascularization

**Figure 3:**
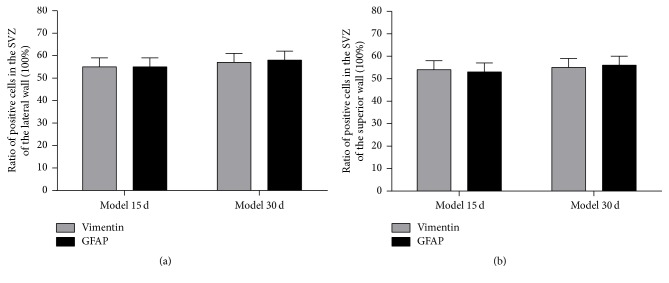
Ratio of glia differentiated from NSCs in the SVZ of the lateral ventricle after cortical devascularization. (a) Ratio of Vimentin-positive cells and GFAP-positive cells in the SVZ of the lateral wall. (b) Ratio of Vimentin-positive cells and GFAP-positive cells in the SVZ of the superior wall.

At 15 days and 30 days after cortical devascularization, the expression of GFAP-positive cells and that of Vimentin-positive cells were both increased in the SVZ of the lateral ventricle. The number of GFAP-positive cells and Vimentin-positive cells both was increased in the SVZ of the lateral wall, superior wall, and medial wall of the anterior corner of the lateral ventricle, especially in the lateral wall. Counting the ratio of glia differentiated from NSCs in the SVZ of the lateral ventricle after cortical devascularization, ratio of glia differentiated from NSCs in the SVZ of the lateral ventricle of devascularized groups at 15 days and 30 days, respectively, was approximately 55% and 57% (Figure 3).

### 3.6. Proliferation and Migration of NSCs in the SVZ in the Migratory Pathway to Cortical Lesions after Cortical Devascularization

**Figure 4:**
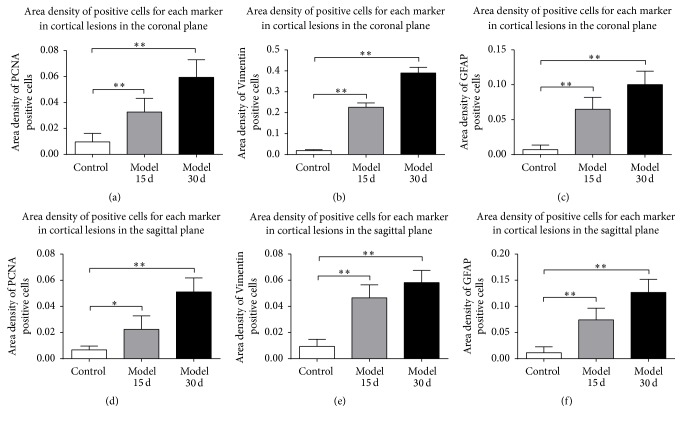
Area density of positive cells in cortical lesions for each marker after cortical devascularization. (a) Area density of PCNA-positive cells in lesions in the coronal plane. (b) Area density of Vimentin-positive cells in lesions in the coronal plane. (c) Area density of GFAP-positive cells in lesions in the coronal plane. (d) Area density of PCNA-positive cells in lesions in the sagittal plane. (e) Area density of Vimentin-positive cells in lesions in the sagittal plane. (f) Area density of GFAP-positive cells in lesions in the sagittal plane. ^*∗*^ *P* < 0.05 and ^*∗∗*^ *P* < 0.01, compared to control group.

**Figure 5:**
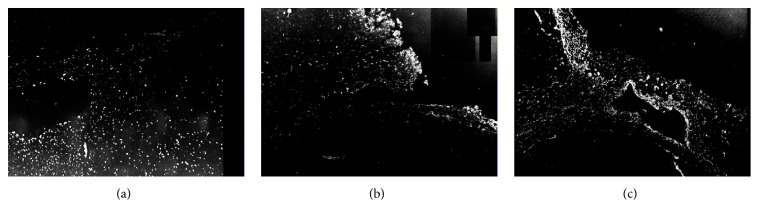
PCNA-, Vimentin-, and GFAP-positive cells in cortical lesions of devascularized groups after cortical devascularization (100x). (a) PCNA-positive cells in lesions site of devascularized groups. (b) Vimentin-positive cells in lesions of devascularized groups. (c) GFAP-positive cells in lesions of devascularized groups.

**Figure 6:**
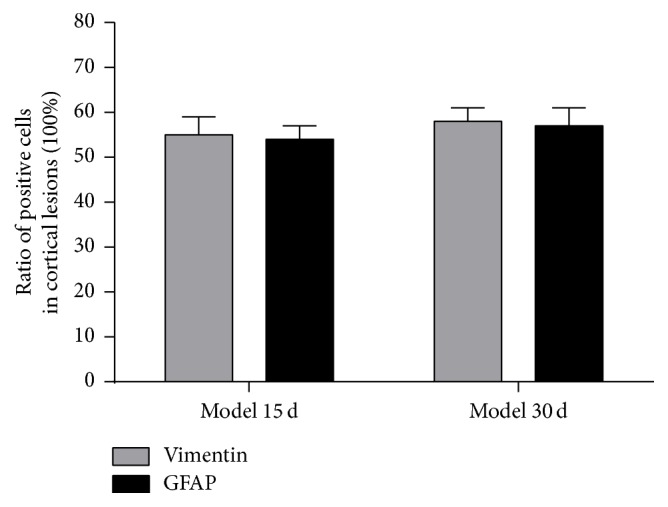
Ratio of glia differentiated from NSCs in cortical lesions of devascularized groups after cortical devascularization.

After cortical devascularization, cells in the SVZ of the damaged side and the contralateral side proliferated and migrated to the corpus callosum, formed a migration path in the corpus callosum, and migrated directly to the cortical lesions. We counted the area density and ran a statistical analysis on the positive cells in the lesion site. Compared with the control group, the area density of PCNA-, Vimentin-, and GFAP-positive cells of devascularized groups in cortical lesions at 15 days and 30 days increased significantly (*P* < 0.05 and *P* < 0.01) (Figures 4 and 5). Counting the ratio of glia differentiated from NSCs in cortical lesions of devascularized groups after cortical devascularization, ratio of glia differentiated from NSCs in cortical lesions of devascularized groups at 15 days and 30 days, respectively, was approximately 55% and 58% (Figure 6).

**Figure 7:**
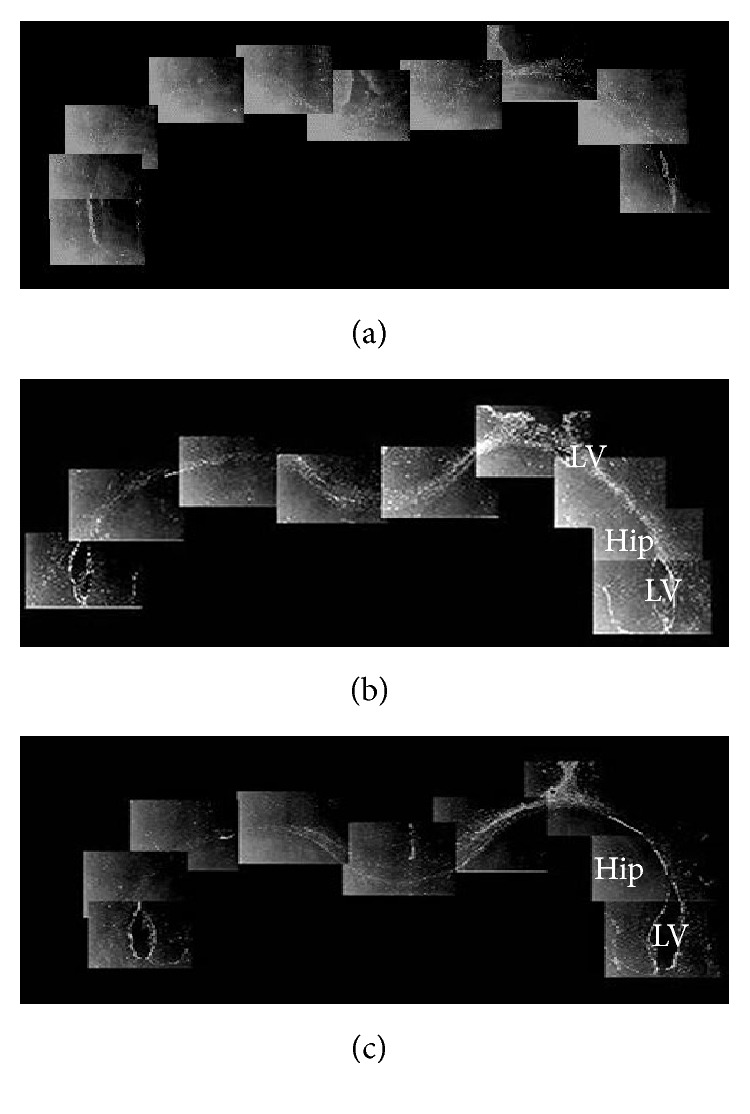
Migratory pathways of PCNA-, Vimentin-, and GFAP-positive cells in the coronal plane (20x). (a) PCNA-positive cells in the SVZ migrated to cortical lesions through the corpus callosum via three migration chains: H, L, and A. (b) Vimentin-positive cells in the SVZ migrated to cortical lesions through the corpus callosum via the same three migration chains. (c) GFAP-positive cells in the SVZ migrated to cortical lesions through the corpus callosum via the same three migration chains. Hip: hippocampus; LV: lateral ventricles.

In normal adult rats, observed from coronal plane, PCNA-, Vimentin-, and GFAP-positive cells were mainly located in the SVZ of the lateral wall of anterior corner, dlC, and LE. Compared with the control groups, the number of PCNA-, Vimentin-, and GFAP-positive cells of devascularized groups increased and had obvious expression in the superior wall (and occasionally the medial wall). These positive cells also migrated from the SVZ to the adjacent part of the corpus callosum. The migration pathway from the dl-SVZ contained three parallel cell bands (L, H, and A) in the corpus callosum of the posterior ventricles (the central portion of the lateral ventricles around the hippocampus and the bottom corner of the SVZ) (Figure 7). The L band arose from the lateral wall of lateral ventricle and traveled along the corpus callosum between the left and right sides of the central corpus callosum. The H band was composed of subependymal cells in the lateral ventricle, which were close to the A band. The A band consisted of hippocampal-type cells. The H chain and the A chain were interconnected in the hippocampal commissure. The three migration chains were comprised of newborn cells marked by PCNA, mature astrocytes marked by GFAP, and immature astrocytes marked by Vimentin. Recent studies indicated that these cells either migrated directly or diffused to lesions and were involved in regeneration and repair [15].

**Figure 8:**
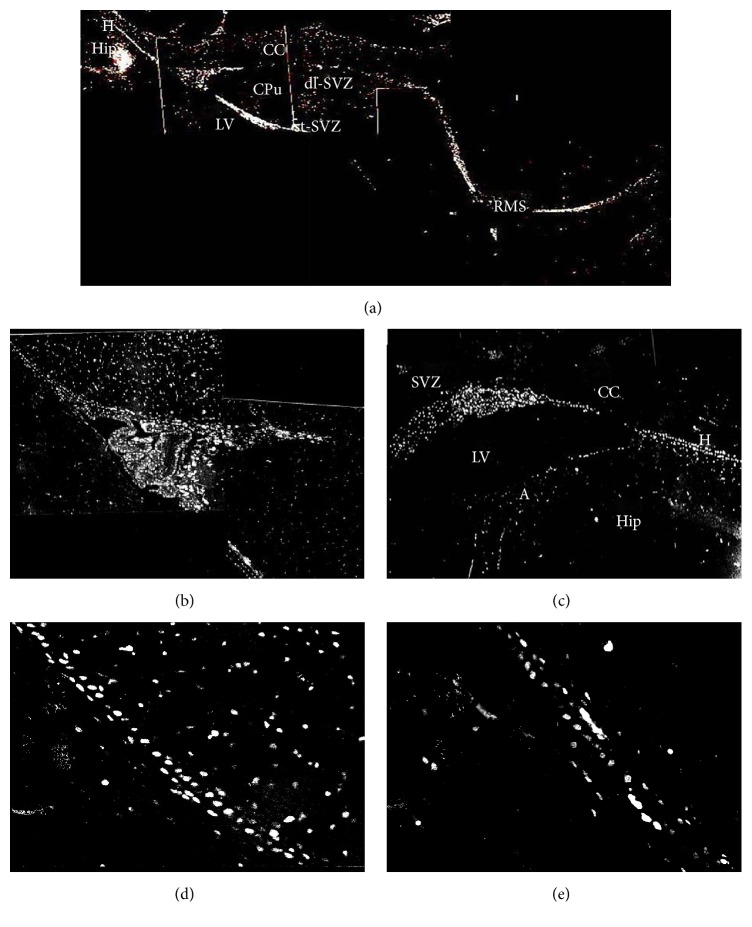
Proliferation and migration of PCNA-positive cells to the lesions in the sagittal plane (20x). (a) PCNA-positive cells formed the RMS and migrated to the olfactory bulb. Some cells diffused through the corpus callosum to the lesions. (b and c) The initial segment of the RMS. (d and e) The middle segment of the RMS. Hip: hippocampus; LV: lateral ventricles; CC: corpus callosum.

**Figure 9:**
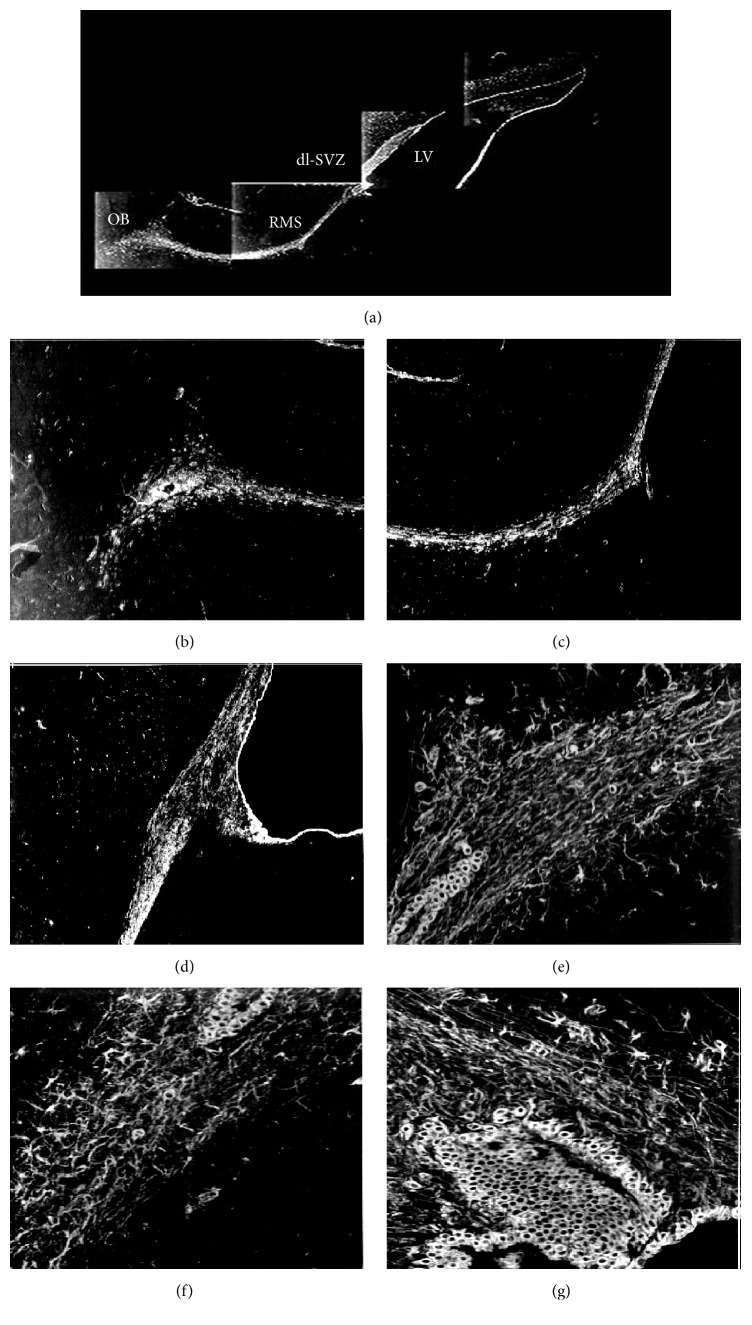
Proliferation and migration of Vimentin-positive cells to the lesions in the sagittal plane. (a) Vimentin-positive cells formed RMS and migrated to the olfactory bulb. Some cells diffused through the corpus callosum to the lesions (20x). (b) The end segment of the RMS (20x). (c) The middle segment of the RMS (20x). (d and g) The initial segment of the RMS (20x). (e) The end segment of the RMS (100x). (f) The middle segment of the RMS (20x).

**Figure 10:**
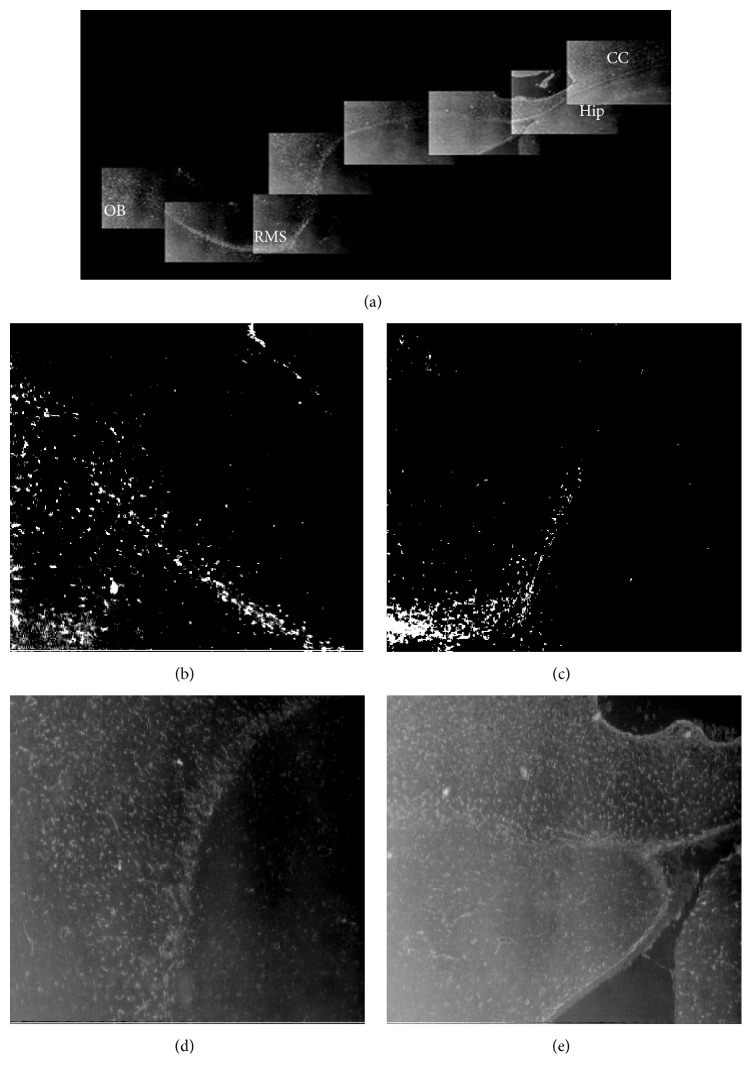
Proliferation and migration of GFAP-positive cells to the lesions in the sagittal plane (20x). (a) GFAP-positive cells formed the RMS and migrated to the olfactory bulb. Some of these cells diffused through the corpus callosum to the lesions. (b) The end segment of the RMS. (c and d) The middle segment of the RMS. (e) The initial segment of the RMS. Hip: hippocampus; CC: corpus callosum.

In the sagittal plane, we clearly observed the full RMS path. Positive cells migrated from the anterior corner of the SVZ (dl-SVZ) obliquely downward between the nucleus accumbens, caudate putamen, and corpus callosum and then forward until they reached the OB and radially diffused into the surrounding tissue. Meanwhile, NSCs in the SVZ also diffused through the corpus callosum to reach the cortical lesions. The above phenomenon was particularly evident when the sections were stained for GFAP and Vimentin (Figures 8, 9, and 10).

### 3.7. Glial Progenitors Derived from the Dorsolateral Subventricular Zone (dl-SVZ) Formed a Migratory Pathway through the Corpus Callosum to the Lesion Site

At 15 days and 30 days after cortical devascularization, Vimentin-, GFAP-, and PCNA-positive cells of the SVZ from the contralateral and ipsilateral dl-SVZ, LE, and dorsal wall of rostral lateral ventricle migrated to the corpus callosum, formed contralateral and ipsilateral migration chains, and radially migrated into the damaged area. The contralateral migration chain migrated toward the ipsilateral hemisphere and across the midline, entering the ipsilateral hemisphere. Positive cells then migrated continuously in the ipsilateral corpus callosum and finally ended in the damage zone. In the contralateral hemisphere, the contralateral migration chains accepted glial progenitor cells from the contralateral dl-SVZ. The migration chains from the ipsilateral hemisphere radiated into the damaged area through the corpus callosum. Positive cells from the ipsilateral dl-SVZ joined the ipsilateral migration chains or migrated directly into the damaged area. We analyzed the chain using serial sections to show that dl-SVZ cells linked together in each plane. Dl-SVZ cells from the contralateral and ipsilateral hemispheres joined the contralateral and ipsilateral migration chains in each coronal section. Migration chains in each section were formed by dl-SVZ cells from many sections (Figure 7).

In the sagittal plane, we observed that PCNA-, Vimentin-, and GFAP-positive cells of the SVZ from the anterior corner of the rostral lateral ventricle formed the RMS to the olfactory bulb, and some of these cells diffused through the corpus callosum to the lesions (Figures 8, 9, and 10).

## 4. Discussion

### 4.1. Proliferation and Glia-Directed Differentiation of NSCs in the SVZ of the Lateral Ventricle after Cortical Devascularization

Studies found that acute injury can promote the proliferation of NSCs in the SVZ such as stroke [17, 18], brain injury [19, 20], and neurodegenerative diseases like Alzheimer's disease [21, 22], Parkinson's disease [23, 24], and experimental autoimmune encephalomyelitis (EAE) [25–28] or epilepsy [29]. The lateral wall of the SVZ contains the highest concentration of NSCs; therefore, most previous studies focused on the influence of cortical injury on the anterior corner of the lateral ventricle, particularly the lateral wall and the RMS. However, the influence of cortical injury on the SVZ of the entire lateral ventricle has not yet been reported. We found that cortical devascularization caused the proliferation of NSCs in the SVZ of the lateral ventricle, including the anterior corner and the inferior corner. The number of PCNA-, Vimentin-, and GFAP-positive cells in the SVZ increased, accompanied by morphological changes that showed an active division phase.

PCNA is a 36-kD protein that is involved in the replication and repair of DNA, which was used to detect proliferating cells. In the S phase of cell proliferation, the expression of PCNA is upregulated. Conversely, the end of cell proliferation and differentiation is accompanied by the downregulation of PCNA expression [30]. Our results showed that after cortical devascularization, the number of PCNA-positive cells was significantly increased in the SVZ of the lateral ventricle, especially in the anterior corner of the lateral wall of the SVZ. PCNA-positive cells were distributed in the anterior corner of the lateral ventricle rod-shaped or in round and oval shapes. Large numbers of PCNA-positive cells were located in the cephalic and the lateral walls of the lateral ventricle. PCNA-positive cells entered the RMS and the corpus callosum from the dlC of the lateral ventricle. Fewer PCNA-positive cells were observed in the ependymal layer than in the SVZ. PCNA-positive cells in the anterior corner of the lateral wall of the SVZ proliferated to form multiple layers and showed split phase. Often, two rod-shaped or oval nuclei existed in pairs, and many cells gathered in clusters. After cortical devascularization, PCNA-positive cells increased, entered the RMS and the corpus callosum from the SVZ, and finally migrated to the cortical lesions.

Doetsch identified cells in the SVZ by morphological and ultrastructural methods and molecular markers. She suggested that cells in the SVZ comprised three different types: nerve cells (cells of type A) forming a migration chain structure, astrocytes (cells of type B) forming a sheath that envelops these chains, and many highly proliferative spherical precursor cells (cells of type C). The network chain made of these three types of cells continues anterior to the lateral ventricle, forming the RMS, terminating in the OB [31, 32]. However, previous findings analyzed the composition of cells in the lateral wall of the normal lateral ventricle, not cells of the superior wall and the medial wall. In this study, we showed that the NSCs in the lateral wall of the SVZ and the superior wall of the lateral ventricle proliferated and formed migration chains to lesions after cortical devascularization.

GFAP-positive cells and Vimentin-positive cells were distributed throughout the rostral to caudal SVZ of the lateral ventricle. More Vimentin-positive cells were observed in the anterior corner and the inferior corner of the SVZ, including the lateral wall, the superior wall, and the medial wall. GFAP-positive cells and Vimentin-positive cells not only increased in the anterior corner of the SVZ and the inferior corner of the lateral wall of the entire lateral ventricle after cortical devascularization but also showed strong expression in the medial wall of the anterior corner. Our results indicated that cortical devascularization caused a rapid proliferation of relatively static NSCs in the SVZ and neurogliocyte increased. The expression of PCNA-positive cells increased, as did the numbers of Vimentin- and GFAP-positive neurogliocytes.

In addition, we observed that GFAP-positive cells and Vimentin-positive cells were found both inside and outside the striatum. Newborn neurons of the lateral ventricle reach the anterior corner of the lateral ventricle, enter the RMS, and continue to migrate forward to the olfactory bulb, where they differentiate into interneurons to replace damaged cells. Studies have shown that in several pathological conditions these cells can migrate out of the RMS to reach to the affected brain areas [33–35]. In this study, we speculated that newborn neurons, which constitute the anterior corner of the RMS, not only came from the lateral ventricle wall but also came from the nearby regions of the lateral ventricle, especially around the anterior corner of the striatum.

### 4.2. Enhanced Proliferation, Differentiation, and Migratory Pathway to the Lesions of NSCs in the SVZ of the Lateral Ventricles after Cortical Devascularization

We found that cortical devascularization induced proliferation and glia-directed differentiation of NSCs on both sides of the dl-SVZ, including PCNA-, Vimentin-, and GFAP-positive cells. The number and the areal density of positive cells in the cortical lesion site increased. Positive cells in the ependymal layer could project to the lesion site, and positive cells in the RMS migrated to the corpus callosum and formed a radial migration pathway to cortical lesions.

Proliferation and migration of NSCs were found in the dl-SVZ in the coronal plane, and these cells also connected with the RMS (rostral) and the SVZ adjacent to the hippocampus (caudal) in the sagittal plane. Dl-SVZ progenitor cells in the RMS formed a tangential migration path in the sagittal plane. In the coronal plane, we observed another path perpendicular to the cells in the sagittal plane. These cells migrated to the dlC from dorsal of the lateral wall and the superior wall of the SVZ and then migrated to the LE, where they formed radial cell migration chains. All parts of the dl-SVZ contained two chains of cells moving in different directions. No matter the direction of these cell chains, they emitted cells that entered the corpus callosum in a perpendicular direction and formed a radial migration path in the corpus callosum. Our results showed that cortical devascularization stimulated NSCs in the SVZ of the lateral ventricle. The number of GFAP-, PCNA-, and Vimentin-positive cells in the SVZ of the lateral ventricle increased significantly accompanied by morphological changes, the area density of positive cells in the cortical lesion site also increased significantly, and these positive cells in RMS increased and formed H, L, and A migration chains and diffused to cortical lesions.

### 4.3. The Essence of the Migratory Pathway of NSCs

It is well known that the development of embryo nervous system originates from the neural plate. The nerve plate is gradually thickened and the front is expanded to a predetermined brain. The neural tube is divided into the forebrain, the midbrain, and the hindbrain before the neural tube closure. After the front of the neural tube is closed, the forebrain gradually forms the telencephalon and the diencephalon and the hindbrain differentiated into the metencephalon and the myelencephalon. Embryos have five cerebral vesicles, namely, the telencephalon, the diencephalon, the mesencephalon, the metencephalon, and the myelencephalon. Finally, the metencephalon differentiated into the cerebellum and the pons and the myelencephalon differentiated into the medulla oblongata. The wall of the cerebral vesicle is gradually thickened and enlarges to both sides, forward and upward, to form the cerebral hemispheres. The cystic rhinencephalon is in the ventral front of the telencephalon, and the rhinencephalon cavity connects with the lateral ventricle. With proliferation and differentiation of the olfactory cells, the rhinencephalon differentiates into the olfactory bulb and the olfactory tract, the lacuna disappearing into a solid structure. The thickening and differentiation of the telencephalon wall are accompanied with the gradual narrowing of the cerebral ventricle, and the morphological changes of both sides of the cerebral ventricle are changed.

In the sagittal plane, we clearly observed the full RMS path. NSCs migrated from the anterior corner of the SVZ to the OB, two layers of cells arranged in order, and parenchyma has thickened. The positive expression of PCNA, Vimentin, and GFAP had characteristics of NSCs, which indicated glia-directed differentiation of NSCs. According to the development of the embryo rhinencephalon, we conjectured that whether RMS was the relic of the rhinocoele closure and whether RMS cells were the neural tube cells left over by the embryo need to be further studied.

As far as dlC was concerned, PCNA-, Vimentin-, and GFAP-positive cells increased in the dl-SVZ, formed a triangle, and migrated outward to the corpus callosum. Two layers of RMS cells arranged in order and parenchyma has thickened. With the rapid development of the telencephalon embryo, the ventricular was gradually narrowed. We conjectured that whether dlC was the relic left over by the narrowed ventricular and whether cells in migration flow were cells in the ventricular zone and the SVZ need to be further studied.

In this study, we showed that PCNA-, Vimentin-, and GFAP-positive cells migrated along the corpus callosum from contralateral and ipsilateral of the lateral ventricles SVZ and formed contralateral and ipsilateral migration chains to damaged area. Brain injury stimulated NSCs in the contralateral and ipsilateral of the lateral ventricles SVZ and promoted proliferation and migration of NSCs to the damaged area. As the channel for the exchange of information on the cerebral hemispheres, migration flow played a critical role in the mechanism of quickly repairing damaged tissue and restoring the brain function.

We observed that PCNA-, Vimentin-, and GFAP-positive cells in the SVZ migrated to the corpus callosum, formed contralateral and ipsilateral migration chains, and radially migrated into the damaged area. NSCs migrated from the corpus callosum with a fan shape and extended to the damaged area. It was indicated that proliferation and glia-directed differentiation of NSCs played a significant role in repairing damaged brain tissue, formed a new nerve ring, and promoted the recovery of brain function.

### 4.4. Molecular Mechanism of Proliferation and Migration of NSCs in the SVZ

NSCs have the capacity of self-renewal, which can generate progenitor cells and other stem cells through asymmetric division. Progenitor cells can divide into neurons and glial cells through limited division, whereas stem cells continue to divide [36]. Studies found that cortical injury can cause the secretion of cytokines and increase the expression of endogenous growth factors in the SVZ and promote the proliferation and differentiation of NSCs in the SVZ, migrating to cortical lesions [37, 38]. High levels of cytokines are produced following acute cortical injury. Excitatory amino acids, calcium ions, free radicals, and cytokines in the extracellular matrix increased significantly in 24 hours after injury, and astrocytes and microglia can be activated to produce cytokines [39]. In the case of brain injury, GFAP- and Vimentin-positive cells can directly or indirectly secrete various cytokines, including bFGF, BDNF, and GDNF. These cytokines promote the differentiation of NSCs into neurons to replace damaged neurons, reestablishing synaptic connections and restoring the original brain function [40]. Recent study also found that neurons in the adult brain tissue cannot regenerate, not because of a lack of stem cells but because of the presence of inhibitory cytokines or the absence of signaling pathways such as mitosis signals and migration signals. The balance between neurotrophic factors and inhibitory factors may determine the regeneration of nervous tissue [41]. In this study, these newborn cells formed certain specific migration paths, including the H, L, and A bands as well as the RMS. Cells migrated directly or through the corpus callosum and then diffused into lesions. The numbers of PCNA-, GFAP-, and Vimentin-positive cells increased in migration paths; the molecular mechanism may be related to secretion of cytokines and increase in the expression of endogenous growth factors and needs to be further researched.

In conclusion, we have identified that proliferation, glia-directed differentiation, and migration of NSCs from the SVZ through the RMS or directly to the corpus callosum after cortical devascularization formed obvious migration flows and dispersed to cortical lesions. These may play a significant role in endogenous repair such that these positive cells could replace the damaged nerve cells in the cortical lesion site and to establish the necessary synaptic contacts to restore normal function.
